# Extendable blocking probe in reverse transcription for analysis of RNA variants with superior selectivity

**DOI:** 10.1093/nar/gku1048

**Published:** 2014-11-05

**Authors:** Tho H. Ho, Kien X. Dang, Susanna Lintula, Kristina Hotakainen, Lin Feng, Vesa M. Olkkonen, Emmy W. Verschuren, Tuomas Tenkanen, Caj Haglund, Kaija-Leena Kolho, Ulf-Hakan Stenman, Jakob Stenman

**Affiliations:** 1 Minerva Foundation Institute for Medical Research, Helsinki, 00290, Finland; 2 Haartman Institute, Department of Clinical Chemistry, Biomedicum Helsinki, University of Helsinki and Helsinki University Central Hospital, Helsinki, FI-00029 HUS, Finland; 3 Helsinki University Central Hospital, HUSLAB, Helsinki, 00029 HUS, Finland; 4 Institute for Molecular Medicine Finland, University of Helsinki, Helsinki, FI-00014, Finland; 5 TJT Technologies Oy, Helsinki, 02360 Espoo, Finland; 6 Department of Surgery, Helsinki University Central Hospital, Helsinki, 00029 HUS, Finland; 7 Research Program Unit, Translational Cancer Biology, University of Helsinki, Helsinki, FI-00014, Finland; 8 Haartman Institute, Department of Pathology, University of Helsinki, Helsinki, FI-00014, Finland; 9 Children′s Hospital, University of Helsinki, Helsinki, FI-00014, Finland; 10 Institute for Molecular Medicine Finland, University of Helsinki, Helsinki, FI-00014, Finland; 11 Department of Women's and Children's Health, Karolinska Institutet, Stockholm, SE-17176, Sweden

## Abstract

Here we provide the first strategy to use a competitive Extendable Blocking Probe (ExBP) for allele-specific priming with superior selectivity at the stage of reverse transcription. In order to analyze highly similar RNA variants, a reverse-transcriptase primer whose sequence matches a specific variant selectively primes only that variant, whereas mismatch priming to the alternative variant is suppressed by virtue of hybridization and subsequent extension of the perfectly matched ExBP on that alternative variant template to form a cDNA–RNA hybrid. This hybrid will render the alternative RNA template unavailable for mismatch priming initiated by the specific primer in a hot-start protocol of reverse transcription when the temperature decreases to a level where such mismatch priming could occur. The ExBP-based reverse transcription assay detected BRAF and KRAS mutations in at least 1000-fold excess of wild-type RNA and detection was linear over a 4-log dynamic range. This novel strategy not only reveals the presence or absence of rare mutations with an exceptionally high selectivity, but also provides a convenient tool for accurate determination of RNA variants in different settings, such as quantification of allele-specific expression.

## INTRODUCTION

Single nucleotide polymorphisms (SNPs) and point mutations are the most common types of genetic polymorphism (1), and they have been widely exploited as important markers in diverse biomedical applications (2). Allele-specific polymerase chain reaction (AS-PCR) and modifications thereof, which are based on the inability of a DNA polymerase to extend a mismatched primer, have been popular in detection of single-nucleotide variants due to their robustness and simplicity (3–24). However, AS-PCR usually has a modest selectivity due to considerable cross amplification. Although a better selectivity can be achieved in certain mutation detection assays, it appears to be dependent on the local sequence and the type of polymorphism (25), and thus cannot be generalized to different mutation detection assays. Furthermore, analysis of RNA variants by AS-PCR and existing technologies requires, at the beginning of the procedure, an additional step to non-specifically convert RNA into cDNA prior to DNA-based mutation detection (26–28), which typically involves the steps of target DNA amplification, an allele discrimination reaction and detection of allele-specific products (29).

In contrast to AS-PCR, allele-specific priming during reverse transcription would in principle allow for direct transformation of highly similar RNA variants into distinguishable allele-specific products in a single step, therefore, it has long been expected to be a promising alternative for analysis of RNA variants. However, pioneering investigators showed that reverse transcriptases could initiate primer extension from a mismatched primer with sufficiently high efficiency (30,31), leading to significant level of mismatch priming. Due to this obstacle, allele-specific priming during reverse transcription has until recently been unattainable with appropriate selectivity. A few approaches have been described for reducing mismatch-priming during reverse transcription by using ‘non-extendable’ competitive blocking probes, but such approaches have still suffered from a fairly low selectivity (32), which restricts their broad usefulness in different settings.

In this report, we describe the first strategy employing an ‘extendable’ competitive blocking probe in reverse transcription (ExBP-RT) to achieve allele-specific priming with superior selectivity, which enables accurate detection and quantification of RNA variants. In ExBP-RT strategy, the mutated RNA template is selectively primed during reverse transcription by the mutation-specific primer, which is coupled to a unique signature at its 5′ end to support detection/discrimination of the allele-specific products in the following step. Most importantly, mismatch priming to the more abundant wild-type RNA template is substantially suppressed by the extendable wild-type-specific competitive blocking probe using a hot-start protocol in reverse transcription.

ExBP-RT is a simple, universal strategy for ultrasensitive detection of mutations in RNA samples and moreover, it enables accurate determination of the RNA levels of expressed mutations, which might reflect the functional consequences brought upon the cells or tissues more faithfully than a DNA-based mutation detection assay (33). This strategy will offer a convenient research tool for epigenetic studies to analyze allele-specific gene expression (34,35), RNA editing (36) as well as detection of RNA mutations within viral RNA genomes such as mutations conferring drug resistance in HIV and influenza viruses (20,37).

## MATERIALS AND METHODS

### RNA samples

RNA templates used for assay development and validation have been generated by *in vitro* transcription using DNA templates with sequences corresponding to the wild-type and different mutated variants of the KRAS and BRAF genes. We prepared DNA templates for *in* *vitro* transcription using PCR amplification of synthetic DNA oligonucleotides, with forward and reverse primers targeting ‘underlined’ sequences (Table 1). PCR amplification has been performed with Phusion High-Fidelity DNA polymerase (Finnzyme, Espoo, Finland, now part of Thermo Scientific) and synthetic DNA oligonucleotides were obtained from TAG Copenhagen A/S. Each oligonucleotide was about 100 nucleotides in length, including 20 nucleotides of T7 promoter's sequence (in bold) at the 5′ end (Table 1).

**Table 1.** Sequences of DNA oligonucleotides used as templates to synthesize different RNA variants

Oligos	Sequences with variant nucleotide in *bold italic*(5′-3′)
KRAS wild-type	**TAATACGACTCACTATAGGG**ATGACTGAATATAAACTTGTGGTAGTTGGAGCTGGTGGCGTAGGCAAGAGTGCCTTGACGATACAGCTAATTCAGAATCA
KRAS G12D (GGT>GAT)	**TAATACGACTCACTATAGGG**ATGACTGAATATAAACTTGTGGTAGTTGGAGCTG***A***TGGCGTAGGCAAGAGTGCCTTGACGATACAGCTAATTCAGAATCA
KRAS G12A (GGT>GCT)	**TAATACGACTCACTATAGGG**ATGACTGAATATAAACTTGTGGTAGTTGGAGCTG***C***TGGCGTAGGCAAGAGTGCCTTGACGATACAGCTAATTCAGAATCA
KRAS G12V (GGT>CTT)	**TAATACGACTCACTATAGGG**ATGACTGAATATAAACTTGTGGTAGTTGGAGCTG***T***TGGCGTAGGCAAGAGTGCCTTGACGATACAGCTAATTCAGAATCA
KRAS G12S (GGT>AGT)	**TAATACGACTCACTATAGGG**ATGACTGAATATAAACTTGTGGTAGTTGGAGCT***A***GTGGCGTAGGCAAGAGTGCCTTGACGATACAGCTAATTCAGAATCA
KRAS G12R (GGT>CGT)	**TAATACGACTCACTATAGGG**ATGACTGAATATAAACTTGTGGTAGTTGGAGCT***C***GTGGCGTAGGCAAGAGTGCCTTGACGATACAGCTAATTCAGAATCA
KRAS G12C (GGT>TGT)	**TAATACGACTCACTATAGGG**ATGACTGAATATAAACTTGTGGTAGTTGGAGCT***T***GTGGCGTAGGCAAGAGTGCCTTGACGATACAGCTAATTCAGAATCA
BRAF wild-type	**TAATACGACTCACTATAGGG**TGAAGACCTCACAGTAAAAATAGGTGATTTTGGTCTAGCTACAGTGAAATCTCGATGGAGTGGGTCCCATCAGTTTGAAC
BRAF V600E (GTG>GAG)	**TAATACGACTCACTATAGGG**TGAAGACCTCACAGTAAAAATAGGTGATTTTGGTCTAGCTACAG***A***GAAATCTCGATGGAGTGGGTCCCATCAGTTTGAAC

The PCR products were used to synthesize RNAs by *in* *vitro* transcription with AmpliScribe™ T7, T3 and SP6 High Yield Transcription Kits (Epicentre Biotechnologies) according to manufacturer's instruction. The concentrations of resulting RNA samples were quantified using a NanoVue spectrophotometer (GE Healthcare, Waskesha, WI), and the copy numbers of the different RNA variants were verified using quantitative RT-PCR (Tetro cDNA synthesis kit and SensiFAST™ SYBR No-ROX Kit, Bioline). The primers for these RT-PCR assays were obtained from TAG Copenhagen A/S. The primer sequences are listed in Table 2. The RNA samples corresponding to KRAS and BRAF wild-type transcript sequences, as well as all possible codon 12 KRAS variants and the BRAF V600E (GTG>GAG) mutation were used for the assay development and the determination of the selectivity of given assays.

**Table 2.** Primer sequences used in RT-PCR assays for quantification of total KRAS and BRAF RNA transcripts

RT-PCR assays	Primers	Concen-tration	Sequences (5′-3′)
KRAS	Reverse transcription primer	0.5 μM	AAATGATTCTGAATTAGCTGT
	PCR forward primer	0.5 μM	GACTGAATATAAACTTGTGGTAGTTG
	PCR reverse primer	0.5 μM	TAGCTGTATCGTCAAGGC
BRAF	Reverse transcription primer	0.5 μM	ACTGTTCAAACTGATGGGACCCAC
	PCR forward primer	0.5 μM	AGACCTCACAGTAAAAATAGGTGA
	PCR reverse primer	0.5 μM	GACCCACTCCATCGAGATTTC

Human RNA samples were extracted from formalin-fixed paraffin embedded (FFPE) samples of colorectal cancer tumor tissue using phenol-chloroform extraction (38). These samples were used as examples of utility of the ExBP-RT assay. The use of clinical samples for this purpose was approved by the institutional review board. All RNA samples were quantified with a NanoVue spectrophotometer (GE Healthcare, Waskesha, WI) and diluted to 500 ng/μl in DEPC H_2_O, before the allele-specific reverse transcription reaction.

### ExBP-RT assay

For each analyzed mutation, a mutation-specific primer was designed to target the mutant RNA and a wild-type-specific blocking probe was designed to target the wild-type RNA (Table 3). The mutation-specific primers included a 5′-prime tail that generated a priming site of non-related sequence for the subsequent amplification reactions. Both the mutation-specific primer and the blocking probe were included in each reverse transcription reaction. All components of the cDNA synthesis reactions except the enzyme reverse transcriptase (Tetro Reverse Transcriptase, Bioline, London, UK) were assembled according to the manufacturer's instruction to a 10-μl reaction volume. The reactions were incubated at 65°C for 5′, then cooled down to 50°C before adding reverse transcriptase enzyme to each well. Subsequently, the reaction temperature was decreased by 1°C every 1 min from 50 to 37°C, then increased to 85°C for 5 min to inactivate the enzyme. The cDNA products were stored at −20°C for later analysis.

**Table 3.** Primer and probe sequences for different ExBP-RT assays

ExBP-RT assays	Primers and probes	Sequences (The engineering 5′-tail sequences in bold)	Concen-trations
KRAS G12D (GGT>GAT)	Mutation-specific primer	**GCCGATCAGACGACGACTATTATT**CCATCAGCT	2 μM
	Wild-type-specific blocking probe	GCCACCAGCT	4 μM
KRAS G12A (GGT>GCT)	Mutation-specific	**GCGCCGATCAGACGACGACTTATT**CCAGCAGC	2 μM
	Wild-type-specific	GCCACCAGCT	4 μM
KRAS G12V (GGT>CTT)	Mutation-specific primer	**GCCGATCAGACGACGACTATTATT**CCAACAGCT	2 μM
	Wild-type-specific blocking probe	GCCACCAGCT	4 μM
KRAS G12S (GGT>AGT)	Mutation-specific primer	**GCCGATCAGACGACGACTATTATT**CCACTAGCT	2 μM
	Wild-type-specific blocking probe	GCCACCAGCT	4 μM
KRAS G12R (GGT>CGT)	Mutation-specific primer	**GCCGATCAGACGACGACTATTATT**CCACGAGC	2 μM
	Wild-type-specific blocking probe	GCCACCAGCT	4 μM
KRAS G12C (GGT>TGT)	Mutation-specific primer	**GCCGATCAGACGACGACTATTATT**CCACAAGCT	2 μM
	Wild-type-specific blocking probe	GCCACCAGCT	4 μM
BRAF V600E (GTG>GAG)	Mutation-specific primer	**GCCGATCAGACGACGACTATTATT**GATTTCTCTGTAG	1 μM
	Wild-type-specific blocking probe	BRAF-INERT: AGATTTCACTGTAG	4 μM
		BRAF-PO4: AGATTTCACTGTAG- PO4	4 μM
		BRAF-Atail: AGATTTCACTGTAG-AAAAAA	4 μM

In the KRAS G12D mutation detection assay a non-extendable oligo that hybridizes to a region downstream of the priming site on the RNA template was used to prevent primer extension resulting from non-specific priming of allele-specific RT primers to a wrong locus downstream of the expected priming site. The sequence of this oligo was: 5′-GAATTAGCTGTATCGTCAAGGCACTAAAAAA-3′.

Multiplex ExBP-RT assay contains, in the same reaction of reverse transcription, six different mutation-specific primers targeting all possible KRAS mutations at codon 12 and a common ExBP targeting the wild-type KRAS transcript (please see Table 3 for sequences of primers and probe). The concentrations of primers and probe are the same as those of primers and probe in ‘single-plex’ ExBP-RT assay. Due to the inclusion of many primers and probe in a single reaction of multiplex ExBP-RT, the optimal concentration of Mg^2+^ ion has been adjusted to 10 mM. In addtition, the unbound primers and nucleotides were degraded using 10 unit of Exonuclease I (Thermo Scientific) and 1 unit of Thermosensitive Alkaline Phosphatase (Thermo Scientific) before proceeding to the quantitative PCR step.

### Quantitative PCR

One microliter aliquots of each cDNA synthesis product were used as templates in the following 10-μl qPCR reactions (SensiFAST™ SYBR No-ROX Kit, Bioline or SensiFAST™ Probe No-ROX Kit, Bioline for probe-based detection). The PCR reactions were incubated at 95°C for 2 min, then for 42 cycles at 95°C for 5 s, 63°C for 20 s and 72°C for 10 s. In multiplex ExBP-RT, we amplified 1 μl of each cDNA synthesis product in the 10-μl qPCR reactions with QuantiTect SYBR PCR Kits (Qiagen) at 95°C for 15 min, then 45 cycles at 94°C for 15 s and 60°C for 45 s, in order to detect either mutant or total KRAS transcripts. The primer and probe sequences for each qPCR assay are listed in Table 4. All qPCR assays were performed on a LightCycler 480 II Real-Time PCR Instrument (Roche Diagnostics Oy, Finland) using 384-well thermal block. Following SYBR-based qPCR, the specificity of the amplification products was always verified by melting curve analysis. Amplification efficiencies of qPCR assays used in this study were determined to be 100%. All reactions were run in duplicate or higher replication where specified. All replicates went through both ExBP-RT and qPCR steps.

**Table 4.** Primer and probe sequences for PCR step of different ExBP-RT assays

ExBP-RT assays	PCR primers	Concen-trations	Sequences (5′-3′)
KRAS G12D	PCR forward primer	0.6 μM	AGGCCTGCTGAAAATGACTG
probe-based qPCR	PCR reverse primer	0.6 μM	CGATCAGACGACGAC
	Probe	0.4 μM	FAM − ATT + AT + TCCA + TCA + gC + TCC − BHQ1 (N+ stands for LNA)
KRAS mutation	PCR forward primer	0.2 μM	GACTGAATATAAACTTGTGGTAGTTG
SYBR Green I qPCR	PCR reverse primer	0.2 μM	CGATCAGACGACGAC
BRAF mutation	PCR forward primer	0.5 μM	TGAAGACCTCACAGTAAA
SYBR Green I qPCR	PCR reverse primer	0.5 μM	CGATCAGACGACGAC
KRAS mutations	PCR forward primer	0.3 μM	CCTGCTGAAAATGACTGAA
(Multiplex ExBP-RT)	PCR reverse primer	0.3 μM	CGATCAGACGACGAC
Total KRAS transcript	PCR forward primer	0.3 μM	CCTGCTGAAAATGACTGAA
(Multiplex ExBP-RT)	PCR reverse primer	0.3 μM	GCCACCAGCTCCAACTACCACAA

### ASB-PCR assay

Reverse transcription was performed using Tetro Reverse Transcriptase (Bioline, London, UK) according to the manufacturer's instructions in a 10-μl volume with 50 nM of each reverse primer. TaqMan PCR was performed with an RT volume of 1 μl in a 10-μl assay with 1× TaqMan^®^ Universal Master Mix II, no UNG (Applied Biosystems, Foster City, CA), 900-nM primers, 200-nM probe and 3600-nM blocker. Standard TaqMan thermocycling conditions were used: 95°C for 10 min, 40 cycles at 95°C for 20 s, at 60°C for 45 s. The oligonucleotides used for KRAS G12D mutation assays has been selected from the original report (19) as following (assay Mut2.1): Forwar mutation-specific primer: tgtggtagttggagctga; Reverse primer: tgattctgaattagctgtatcgtcaa; Blocker: ttggagctggtggcgtagg-PO4; Taqman probe: FAM − cac + Tc + T + Tgcctacgc − BHQ1 (+N stands for LNA nucleotide). Each ASB-PCR assays were run in duplicate on a LightCycler 480 II Real-Time PCR Instrument (Roche Diagnostics Oy, Finland).

### Data analysis

Threshold cycle (Ct) values were calculated automatically using the default second derivative maximum method, which is built in the LightCycler^®^ 480 II system (Roche Diagnostics Oy, Finland).

The selectivity of each ExBP-RT or other assays for detecting mutant RNA transcripts among a surplus of wild-type transcripts is determined by comparing products formed in the first reaction containing mismatched template (wild-type RNA) with those formed in the second reaction containing the same copy number (10^7^ copies) of matched templates (mutant RNA). The ratio of product formed in the first reaction normalized to product formed in the second reaction can be determined by quantitative PCR based on the difference in Ct values derived from two reactions (ΔCt_wt-mt_ = Ct_wild-type_ − Ct_mutant_). The selectivity of each ExBP-RT assay expressed as percentage was calculated as 2^−ΔCt^ × 100%, which correspond to the lowest fraction of mutant transcripts to be detected as a distinct signal from the background signal derived from the wild-type template.

## RESULTS

### Overview of ExBP-RT strategy

**Figure 1.**
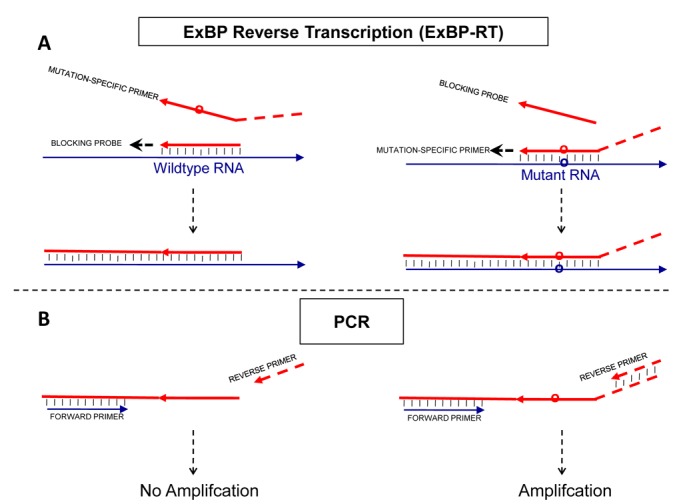
Principle of the allele-specific reverse transcription (ExBP-RT) assay. The analytical procedure includes two steps: (**A**, step 1) reverse transcription with a mutation-specific RT primers and an extendable competitive blocking probe (**B**, step 2) selective PCR amplification and detection/quantification. While the mutation-specific primer, which contains a nucleotide tail of unrelated sequence, generates a PCR-amplifiable cDNA product, the competitive blocking probe without tail produces a cDNA lacking primer-binding site for the reverse PCR primer.

We established the ExBP-RT strategy for direct transformation of RNA variants with minor differences, such as single-base substitutions, into easily distinguishable allele-specific products with superior selectivity using a single reaction of reverse transcription. The reverse transcription reaction contains a mutation-specific primer and a wild-type-specific competitive blocking probe that is extendable (Figure 1). Both mutation-specific RT primer and the blocking probe contain a priming sequence that is fully complementary to a specific RNA variant, but forms a mismatch with the alternative RNA variant at the variation site. The priming sequences are designed to be short (∼10 nucleotides in length and Tm ≅ 37–50°C, according to DINAMelt web server (39), which is based on the unified nearest-neighbor parameters of oligonucleotides (40)), in order to maximize the discrimination between perfect match and mismatch. By utilizing a hot-start protocol in reverse transcription, with a slow cool down step toward the optimal temperature for primer annealing, the correct priming occurs at substantially higher temperature and temporally prior to mispriming. In particular, the mutated RNA template is selectively primed by the mutation-specific primer during reverse transcription and the resulting cDNA product is detected in the following step based on a unique signature coupled to the 5′ end of mutation-specific primer. Mismatch priming of the mutation-specific primer is suppressed by neutralizing the wild-type template in the beginning of the reaction with an extendable blocking probe. This is done by hybridization and extension of the perfectly matched competitive blocking probe on the wild-type template. The forming cDNA binds to the wild-type RNA template with a far greater affinity than the original blocking probe does, thus efficiently blocking the possible site for mismatch priming caused by mutation-specific primer, when the temperature is decreased to a level where mispriming would be likely to occur.

In the present report, the unique signature coupled to the 5′ end of mutation-specific primer is a tail of unrelated sequence, which forms a unique downstream priming site in the mutation-specific cDNA product. This distinction between the mutation-specific cDNA product and blocking cDNA allows selective amplification and detection/quantification of the mutation-specific cDNA products by quantitative PCR, while the wild-type cDNA that lacks the primer-binding site for the reverse PCR primer (Figure 1B), will not be amplified.

### Detection of mutant KRAS G12R RNA in mixtures with excessive amounts of KRAS wild-type RNA

**Figure 2.**
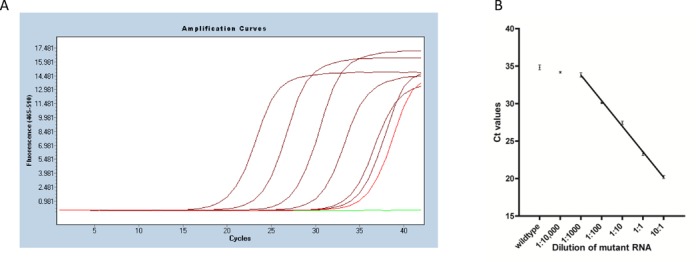
Detection of mutant KRAS G12R RNA with the ExBP-RT assay. (**A**) Representative qPCR amplification curves of mutant KRAS G12R RNA serially diluted into wild-type KRAS RNA (from left to right) 10:1, 1:1, 1:10, 1:100, 1:1000 and 1:10,000 (in dark red), wild-type KRAS RNA only (in light red) and H2O control (in green). (**B**) The mean Ct values (three independent assays) were plotted against the dilution of mutant RNA.

To demonstrate proof of principle, we prepared several RNA mixtures of two *in* *vitro*-transcribed KRAS RNA variants with different ratios. 10^7^ copies of wild-type KRAS RNA were mixed with various copies of mutant KRAS G12R RNA (10^8^ to 10^3^ copies), so that the ratio of mutant versus wild-type KRAS RNA ranged from 10 down to 10^−4^ (10, 1, 10^−1^, 10^−2^, 10^−3^, 10^−4^). The assays were performed using the ExBP-RT strategy followed by SYBR Green I-based quantitative PCR. Details on reaction conditions are described in the ‘Materials and Methods’ section. Mutant KRAS G12R RNA was detected as a signal distinct from the wild-type KRAS RNA at least in mixtures containing a proportion of 1:1000 or more of mutant KRAS G12R RNA (Figure 2a) and the detection was linear from 1:1000 to 10:1 (Figure 2b, *r*^2^ = 0.9955, least-squares analysis). The superior selectivity of the assay has also been verified using various copies (10^8^ to 10^3^ copies) of wild-type KRAS RNA only or mutant KRAS G12R RNA only as template (Supplementary Figure S1). The amplification products have been visualized on agarose gel 2% in order to confirm the expected size (59 bp) of amplified sequences (Supplementary Figure S2). Omission of RNA template, mutation-specific primer or reverse transcriptase in negative control samples leads to complete lack of amplification.

### Detection of all six possible KRAS mutations at codon 12 and the BRAF V600E mutation from RNA samples

The applicability and selectivity of the ExBP-RT strategy for analysis of RNA variants was assessed on a panel of different templates and mutation types to detect all six possible mutations at codon 12 of the KRAS gene and the BRAF V600E mutation. The primer sequences for each assay are listed in Table 3. The selectivity of each ExBP-RT assay was determined by comparing products formed in the first reaction containing mismatched template with those formed in the second reaction containing the same copy number of matched templates (Please see ‘Materials and Methods’ section for more detail).

The selectivity expressed as percentage of each ExBP-RT assay (2^−ΔCt^ × 100%) was shown in Table 5. The ExBP-RT assays was able to detect different mutant KRAS or BRAF RNAs in the presence of 1000 to 6000-fold more excessive background of wild-type transcripts at a selectivity ranging from 0.017 to 0.09%.

**Table 5.** The selectivity of ExBP-RT assays to detect different KRAS mutations at codon 12 and the BRAF V600E mutation

Mutations	ΔCt_wt-mt_	Selectivity (%)
KRAS G12D (GGT>GAT)	11.4 ± 0.16	0.04%
KRAS G12A (GGT>GCT)	10.5 ± 0.05	0.07%
KRAS G12V (GGT>CTT)	11.3 ± 0.09	0.04%
KRAS G12S (GGT>AGT)	10.2 ± 0.10	0.09%
KRAS G12R (GGT>CGT)	12.5 ± 0.13	0.017%
KRAS G12C (GGT>TGT)	12.2 ± 0.07	0.021%
BRAF V600E (GTG>GAG)	12.5 ± 0.05	0.017%

### Comparison of ExBP-RT strategy to existing technologies

In order to verify the performance of the competitive extendable blocking probe in reverse transcription, we conducted a comparison to different existing approaches for analysis of single-nucleotide RNA variants.

**Figure 3.**
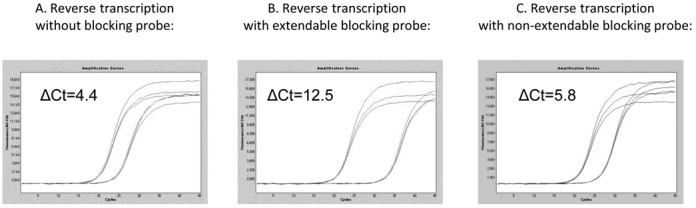
qPCR amplification curves (three replicates) derived from the same copy number of either mutant transcripts (left curves) or wild-type transcripts (right curves). cDNA synthesis reactions were performed either in the absence of competitive blocking probe (**A**), in the presence of an extendable competitive blocking probe (**B**) or in the presence of non-extendable competitive blocking probe (**C**).

In the first set of experiment, BRAF V600E mutation detection assay has been selected as a model to compare the ExBP-RT strategy with different approaches to achieve allele-specific priming during reverse transcription. All reverse transcription reactions share the same thermal program, reagents and BRAF mutation-specific primer, but each assay contains different blocking probes or does not contain any blocking probe. While the ExBP-RT assay based on an extendable competitive blocking probe gave rise to a ΔCt_wt-mt_ of 12.5 corresponding to a selectivity of 0.017% (Figure 3b), the reverse transcription without any blocking probe results in a ΔCt_wt-mt_ of only 4.4 (Figure 3a), corresponding to a significantly lower selectivity of 5% (= 1/2^4.4^ × 100%). When a non-extendable blocking probe is added to the reverse transcription reaction, the resulting ΔCt_wt-mt_ increases modestly to 5.8 (Figure 3c), which corresponds to a selectivity of about 1.8%. Although this selectivity is improved in comparison to that of the assay not containing any blocking probe, it is 100× lower than that achieved by the ExBP-RT strategy. This data clearly shows that an extendable wild-type-specific blocking probe outperforms the non-extendable alternative for suppression of the mismatch priming during reverse transcription. This subsequently leads to a superior selectivity of the ExBP-RT assay for analysis of RNA variants.

Unlike almost all existing technologies for discrimination of RNA variants, which utilize a DNA-based allele discrimination reaction going after a ‘non-specific’ reverse transcription, the selectivity of ExBP-RT is attributed directly to allele-specific priming at the stage of reverse transcription. In addition to the contrast in methodology, we further compare the selectivity of the ExBP-RT assay with that of other existing technologies in the second set of experiments. KRAS G12D (GGT>GAT) mutation has been selected as model, because ExBP-RT assay for detection of this RNA variant involve the most stable primer:template mismatch (dT:rG) (41). Different AS-PCR assays described in a recent paper (19) for detection of this RNA variant was adopted for this comparative experiment. While the ExBP-RT assay for detection of mutant KRAS G12D RNA has a selectivity of 0.04% as shown above, the selectivity of corresponding optimal ASB-PCR assay (originally named Mut2.1 assay), which employs both low-Tm discriminating PCR primer and blocking probe, was only 0.15%. Although the ΔCt_wt-mt_ value of this ASB-PCR assay was 10.1 (Supplementary Figure S3B), which is in line with the original report, this translated into a relatively low selectivity due to the low PCR amplification efficiency of only 90% (Supplementary Figure S3A). The PCR primer with low Tm and the inhibiting effect of blocking probe probably compromise the PCR amplification efficiency in this ASB-PCR assay. When the blocking probe is excluded from the Mut2.1 assay, the selectivity of consequent AS-PCR assay is greatly attenuated to 20% (2^−2.3^ × 100%), which is also consistent with the original report. It is worthy noted that this Mut2.1 assay involve the primer:template mismatch (dA:dC), which is reasonably unstable. When the discriminating primer was designed to target the same mutation but on the other strand (originally named Mut2.2 assay), the most stable primer:template mismatch (dT:dG) is involved and the selectivity of the assay is reported to be even worse (19).

### Detection of mutant KRAS G12D RNA in FFPE samples

**Figure 4.**
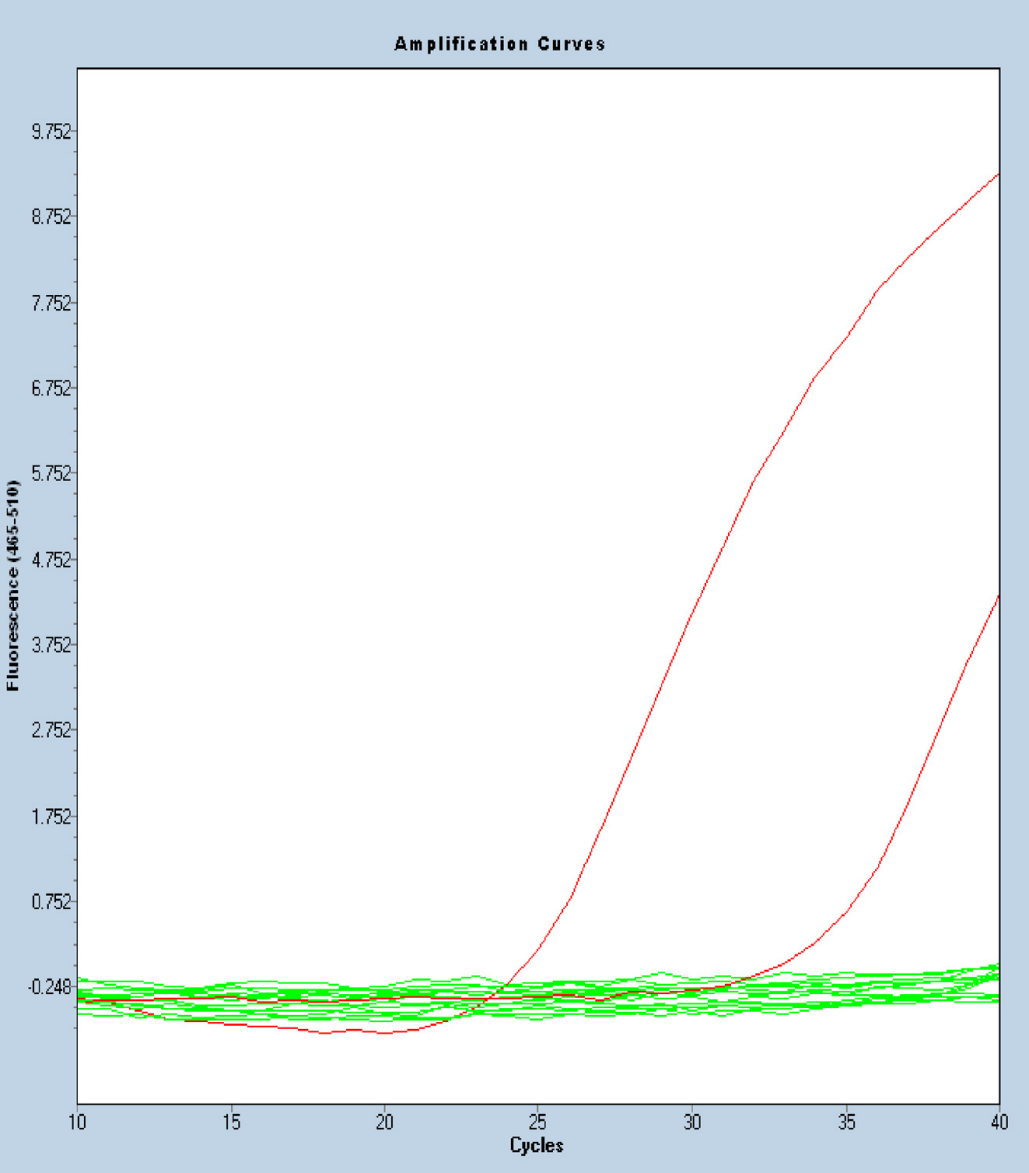
Detection of mutant KRAS G12D RNA in FFPE samples from colorectal cancer patients. qPCR amplification curves of ExBP-RT assay for detection of mutant KRAS G12D RNA in 11 FFPE samples of colorectal cancer patients.

The PCR step of the above examples utilizes SYBR Green I detection, but quantitative PCR using sequence-specific probes can equally be used for detection following allele-specific priming during reverse transcription. The following example of utility illustrates such a probe-based assay to detect the most common KRAS mutation type (G12D), which accounts for ∼32.5% of all KRAS mutations (42), from FFPE samples of colorectal cancer patients. The reverse transcription was performed as described with primer and probe sequences listed in the Table 3. Identical amount of RNA (500 ng) extracted from 11 FFPE samples was used as a template for the allele-specific reverse transcription reactions. One out of 11 samples was clearly positive for the G12D mutation (Figure 4) with a mean Ct value of 34.5 (right red curve). The positive control containing mutant RNA (10^7^ copies) show early amplification (left red curve), whereas omission of RNA template or reverse transcriptase in negative controls leads to complete lack of amplification. All tested clinical samples were shown to contain comparable amounts (0.6 – 1.8 × 10^5^ copies/500 ng of total RNA) of total KRAS RNA (Supplementary Figure S4), which are sufficient for the assay reproducibility (43). Agarose gel plot also confirmed the specific band of expected size for the tested positive sample and no specific band detected for all the tested negative samples (Supplementary Figure S5)

### Multiplex detection of different KRAS mutations at codon 12

**Figure 5.**
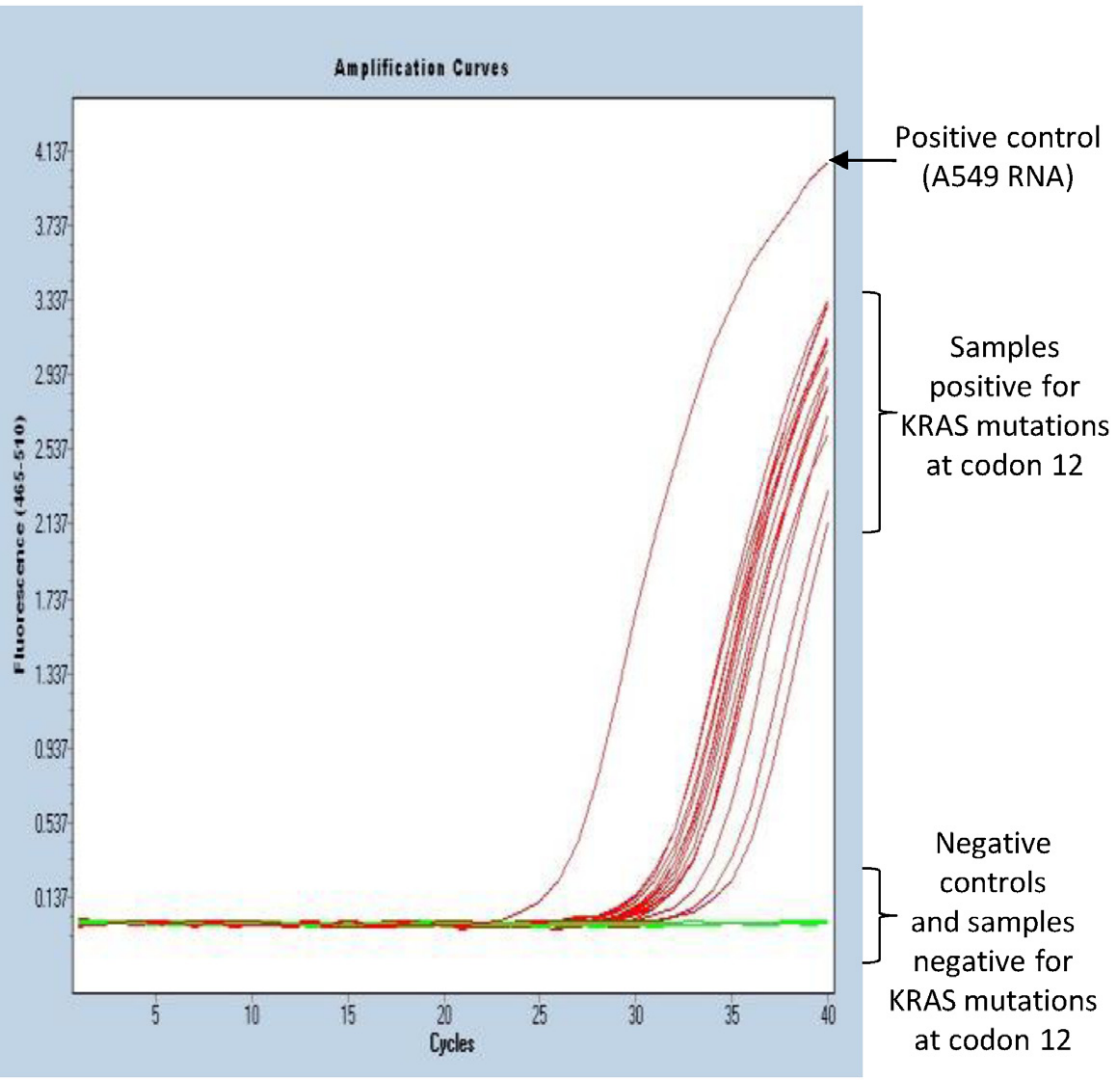
Simultaneous detection of all six possible KRAS mutations at codon 12 in FFPE samples from colorectal cancer patients. Representative qPCR amplification curves of multiplex ExBP-RT assay for detection of KRAS mutations at codon 12 in 44 FFPE samples of colorectal cancer patients.

Because the ExBP-RT assay is, unlike AS-PCR, performed during a single cycle, the common problems with formation of primer-dimers in multiplex PCR assays is completely avoided. Hence, designing a multiplex assay is straightforward, and simultaneous detection of several mutations in the same reaction tube can be easily achieved. The following example of utility describes such a multiplex ExBP-RT assay to detect all six possible mutations within codon 12 of KRAS gene, including KRAS G12A, G12C, G12D, G12R, G12S and G12V mutations. In contrast to ‘single-plex’ assays mentioned above, the multiplex ExBP-RT assay contains, in the same reaction of reverse transcription, six different mutation-specific primers targeting all possible KRAS mutations at codon 12 and a common ExBP targeting the wild-type KRAS transcript (please see Table 3 for sequences of primers and probe). Identical amount of RNA (500 ng) extracted from 44 FFPE samples of colorectal cancer patients has been used as templates for this multiplex ExBP-RT assay. Positive controls containing total RNA extracted from KRAS-mutant cell line A549 show early amplification, and 16 out of 44 samples (36.4%) were clearly positive for KRAS mutations at codon 12 (Figure 5). All tested mutation-positive and mutation-negative clinical samples were shown to contain comparable amounts of total KRAS RNA (Supplementary Figure S6). There was no amplification in negative controls containing either H_2_O or RNA extracted from KRAS-wild-type cell line COLO205, and omission of RNA template or reverse transcriptase also lead to complete lack of amplification. The mutation rate observed in archival FFPE samples from patients with colorectal cancer was slightly higher than what has been reported (27.7%) in a worldwide multicenter study using DNA-based mutation detection assays (44). When analyzing another series of 87 RNA samples derived from colonoscopy biopsies of paediatric patients with non-malignant conditions, using the same multiplex ExBP-RT assay, no KRAS mutations were detected at codon 12 (unpublished data). In this series wild-type KRAS was readily detected in all samples.

## DISCUSSION

In this paper we show that (i) extendable competitive blocking probes enable allele-specific priming during reverse transcription with superior selectivity, particularly when utilizing a hot start and subsequent slow cool down temperature protocol; (ii) extendable wild-type-specific blocking probes outperform non-extendable substitutes to suppress mismatch priming during reverse transcription and (iii) an engineered 5′-tail sequence in the mutation-specific RT primer allows for selective PCR amplification/enrichment of the rare variant, completely avoiding the amplification-dependent decay of selectivity which is a well known problem related to allele-specific PCR. This ultimately results in convenient assay for ultrasensitive detection of mutant RNA in the presence of a 1000–10 000-fold excess of the wild-type counterpart.

Currently, various strategies have been established for SNP genotyping and mutation detection, usually containing the steps of target DNA amplification, allele discrimination reaction and detection/identification of allele-specific products (29). The target amplification is performed as the first step in almost all current strategies (2), but there are several limitations associated with this approach. For example, when the target amplification step is performed prior to a separate allele discrimination reaction, such as mini-sequencing (45–47), it is obligatory to handle post-amplification products, which increases the risks of laboratory contamination. Furthermore, since both mutant and wild-type variants are amplified with the same efficiency, the PCR product resulting from a rare variant will not be enriched, and may thus be below the limit of detection, even when the amplification is in the plateau phase. On the other hand, when the target amplification step coincides with the allele discrimination reaction, such as in allele-specific PCR, the allele discriminating power decays quickly during the course of the amplification process (29,48).

In the ExBP-RT strategy presented here, the allele discrimination reaction takes place during reverse transcription; accordingly the requirement of either performing a separate step for allele discrimination or handling of post-amplification products is avoided. Since the reverse transcription reaction is performed during a single cycle, the reaction time is significantly longer than the annealing and extension times typically used in PCR. This allows for optimizing the primers solely for allele discrimination, without any restrictions or compromises that arise from concurrent optimization to improve PCR performance. In particular, the length of the target-specific segment of the mutation-specific primers and blocking probe used in ExBP-RT is typically only about 10 nucleotides (Tm ≅ 37–50°C), which allows for optimal discrimination between mutant and wild-type variants in addition to assure high specificity of the priming during reverse transcription reaction. It is worthy noted that the Tm values of those primers and probes are sufficiently high for our ExBP-RT assay, where the lowest temperature of the RT protocol is 37°C. Most importantly, since the allele discrimination reaction takes place prior to selective amplification of the target cDNA products, the allele discriminating power achieved during reverse transcription is unaffected by the exponential PCR amplification as opposed to allele-specific PCR.

Non-extendable wild-type-specific blocking probes have been widely used to reduce mispriming, and at the same time, to improve the selectivity of most previously described competitive allele-specific PCR assays (6,8,15,16,18,19,23,24,49,50). These probes suppress mispriming of the mutation-specific primers by blocking the potential priming site upon hybridization to the wild-type template. Our data shows, however, that the extendable wild-type-specific blocking probes exhibit superior performance on suppression of mispriming during reverse transcription compared to non-extendable substitutes. The underlying mechanism of action resulting in this difference involves initiation of primer extension to form cDNA upon hybridization of ExBP to the wild-type template. This wild-type cDNA–RNA hybrid is much more thermostable than hybridization of any non-extendable oligonucleotide probe due to the significantly greater length and therefore, efficiently blocks the possible mispriming sites for the mutation-specific primers as the reaction temperature is gradually decreased. This ultimately results in effective avoidance of mispriming to abundant wild-type RNA template and dramatically improves the selectivity of the ExBP-RT assay.

Unlike allele-specific PCR where stringent optimization of PCR reaction conditions is often required to achieve sufficient sensitivity (3–5,9,11,17,21,51–53), ExBP-RT assays are relatively simple to set up without massive optimization. In particular, all major components of the cDNA synthesis reaction are within the general recommendations of the manufacturers and a universal protocol can be applied for detection of all mutation types on different genes. Furthermore, the performance of ExBP-RT assays is likely to be less influenced by the nature of polymorphisms and surrounding sequences in comparison to AS-PCR (29). Although additional evidence analyzing different mutations in a broader set of genes is required, our study shows that the sensitivity of different assays varies within a narrow range (10^−3^ – 10^−4^). Even the ExBP-RT assays for KRAS G12D (GGT>GAT) and KRAS G12S (GGT>AGT) mutations, which involve the most stable primer:template mismatch (dT:rG) (41), exhibit sufficiently good selectivity of 4 × 10^−4^ and 9 × 10^−4^, respectively. Another important feature of the ExBP-RT assay is the enrichment of mutations to facilitate detection of rare mutant alleles (54), as only mutant cDNA product comprising the engineered 5′-tail sequence is amplified during PCR. For the purpose of clarification, this manuscript demonstrated ExBP-RT assays for detection of mutant RNA variants in an excessive background of wild-type counterparts; however, the technique can be generally applied to detect any RNA variant in the presence of the alternative variant regardless of their relative abundance. As an example, the ExBP-RT assay for detection of mutant KRAS G12D RNA in the presence of another mutant variant, KRAS G12A RNA, used as a wild-type surrogate, exhibited an excellent selectivity of 0.05% with a ΔCt_wt-mt_ of 11.01 cycles (Supplementary Figure S7).

This technique is clearly in its infancy and our results must be interpreted in the context of a number of potential limitations. While there are several reports of modified nucleotides that significantly improve the discrimination between matched and mismatched primer:template duplexes (51,52,55–58), the effects of different nucleotide modifications on the selectivity of ExBP-RT assays have not been tested in this study. Although the use of modified nucleotides increases the cost of testing, studies to systematically investigate the benefits of different modified nucleotides are warranted to boost up the selectivity of this novel method even further for certain applications (59,60). In addition, we have established ExBP-RT assays for detection of only seven different mutations in this study, which does not cover all possible mutation types. Consequently, more ExBP-RT assays need to be set up in order to draw more reliable conclusions about the selectivity and applicability of this technique in comparison with other currently accepted strategies of mutation detection.

In conclusion, we have established a novel strategy for analysis of RNA variant with superior selectivity using extendable competitive blocking probe in reverse transcription (ExBP-RT). This technique is valuable for mutation detection with ultra-high selectivity and for analysis of expressed mutations at the RNA level, which might, more faithfully reflect the functional consequences brought upon the cells or tissues. ExBP-RT is especially useful in situations where RNA is a mandatory starting material, such as in epigenetic studies to analyze allele-specific gene expression, miRNA variants, exosomal RNA variants or RNA editing and detection of mutations within the genome of RNA viruses. The analytical procedure of ExBP-RT is rapid and requires only moderate optimization. In combination with qPCR using intercalating dyes or probe-based detection, ExBP-RT is a sensitive strategy for mutation detection from RNA or for analysis of RNA variants. Although not examined in depth in this study, it is reasonable to assume that the ExBP-RT strategy might also support detection and discrimination of highly similar transcript variants in single cells in a solution-based or *in* *situ* platform. The demand for this type of analysis was highlighted in two recent papers describing methodology for allele-specific detection of individual mRNAs (61,62).

## SUPPLEMENTARY DATA

Supplementary Data are available at NAR Online.

SUPPLEMENTARY DATA
