# Supplementary material for: Extendable blocking probe in reverse transcription for analysis of RNA variants with superior selectivity
Source: Nucleic Acids Res. 2014 Nov 5;43(1):e4. doi: 10.1093/nar/gku1048 (PMC4288146; doi:10.1093/nar/gku1048)
Supplement: SUPPLEMENTARY DATA [file supp_43_1_e4__index.html]

Extendable blocking probe in reverse transcription for analysis of RNA variants with superior selectivity — Extendable blocking probe in reverse transcription for analysis of RNA variants with superior selectivity — SUPPLEMENTARY DATA 

# Extendable blocking probe in reverse transcription for analysis of RNA variants with superior selectivity

## SUPPLEMENTARY DATA

**Files in this Data Supplement:**

- SUPPLEMENTARY DATA
