# Supplementary material for: Extendable blocking probe in reverse transcription for analysis of RNA variants with superior selectivity
Source: Nucleic Acids Res. 2014 Nov 5;43(1):e4. doi: 10.1093/nar/gku1048 (PMC4288146; doi:10.1093/nar/gku1048)
Supplement: SUPPLEMENTARY DATA [file supp_gku1048_nar-02599-met-g-2014-File007.pdf]

SUPPLEMENTARY DATA

A

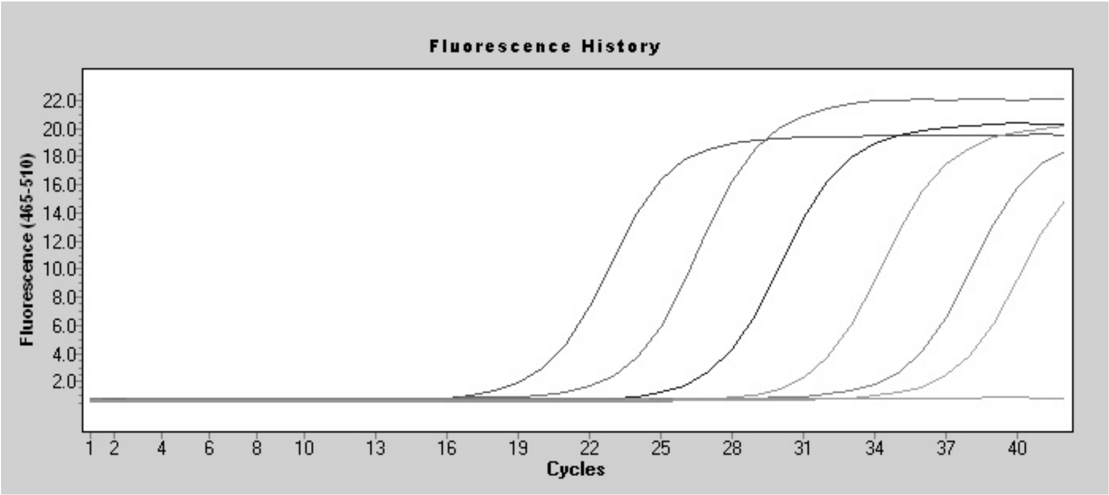

B

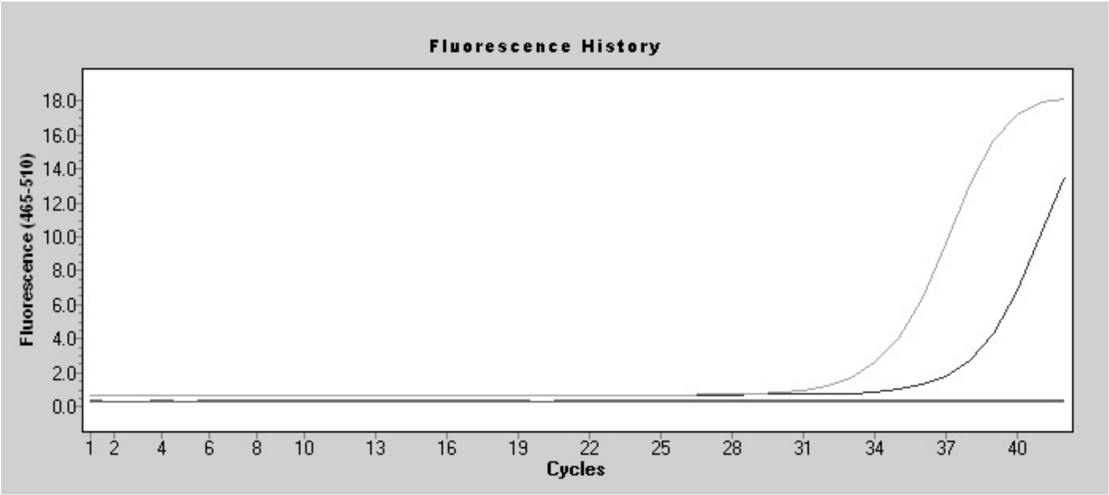

Figure 1S. Representative amplification curves of ExBP-RT assay using single RNA templates. (A) qPCR amplification curves of mutant KRAS G12R RNA only (from left to right) 10^8, 10^7, 10^6, 10^5, 10^4, 10^3, H_2O. (B) qPCR amplification curves of wildtype KRAS RNA only (from left to right) 10^8, 10^7. An extendable blocking probe was used in reverse transcription to suppress amplification of the wildtype KRAS RNA template. Due to cross-reactivity in reverse transcription, some amplification occurred in samples containing more than 10^7 copies of wildtype template. Samples containing 10^6 copies or less of wildtype KRAS RNA show no amplification.

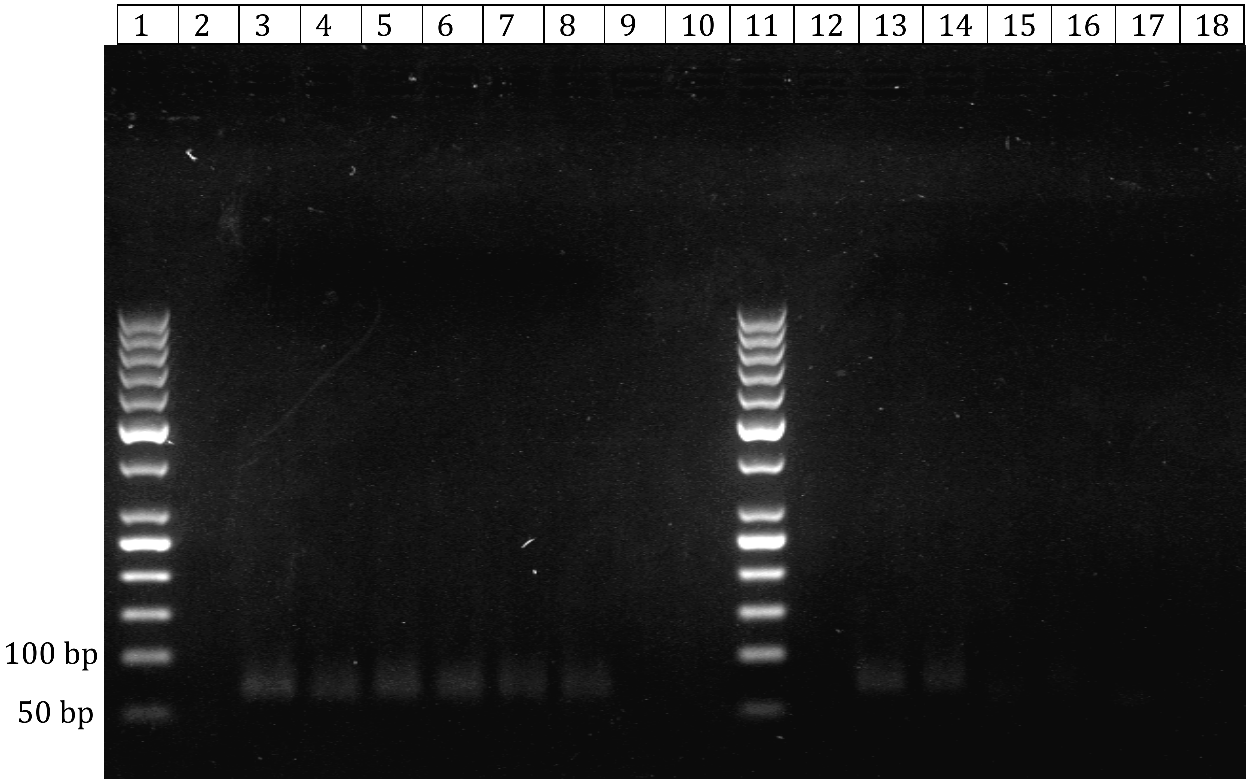

Figure 2S. Visualization of ExBP-RT amplification products on agarose gel 2%. The expected size (59 bp) of specific amplified sequences has been confirmed after 42 cycles of PCR for all ExBP-RT assays containing 10^8, 10^7, 10^6, 10^5, 10^4, 10^3 copies of mutant KRAS G12R RNA (lanes 3-8, respectively) and those assays containing 10^8, 10^7 copies of wildtype KRAS RNA (lanes 13,14, respectively). No specific amplification product has been detected on gel for assays containing lower copy numbers of wildtype KRAS RNA (10^6, 10^5, 10^4, 10^3 copies, corresponding to lanes 15,16,17,18) or H_2O (lane 9). Lanes 1 and 11 are GeneRuler 50bp DNA ladder, ready-to-use (Thermo Scientific). Lanes 2,10 and 12 are blank wells.

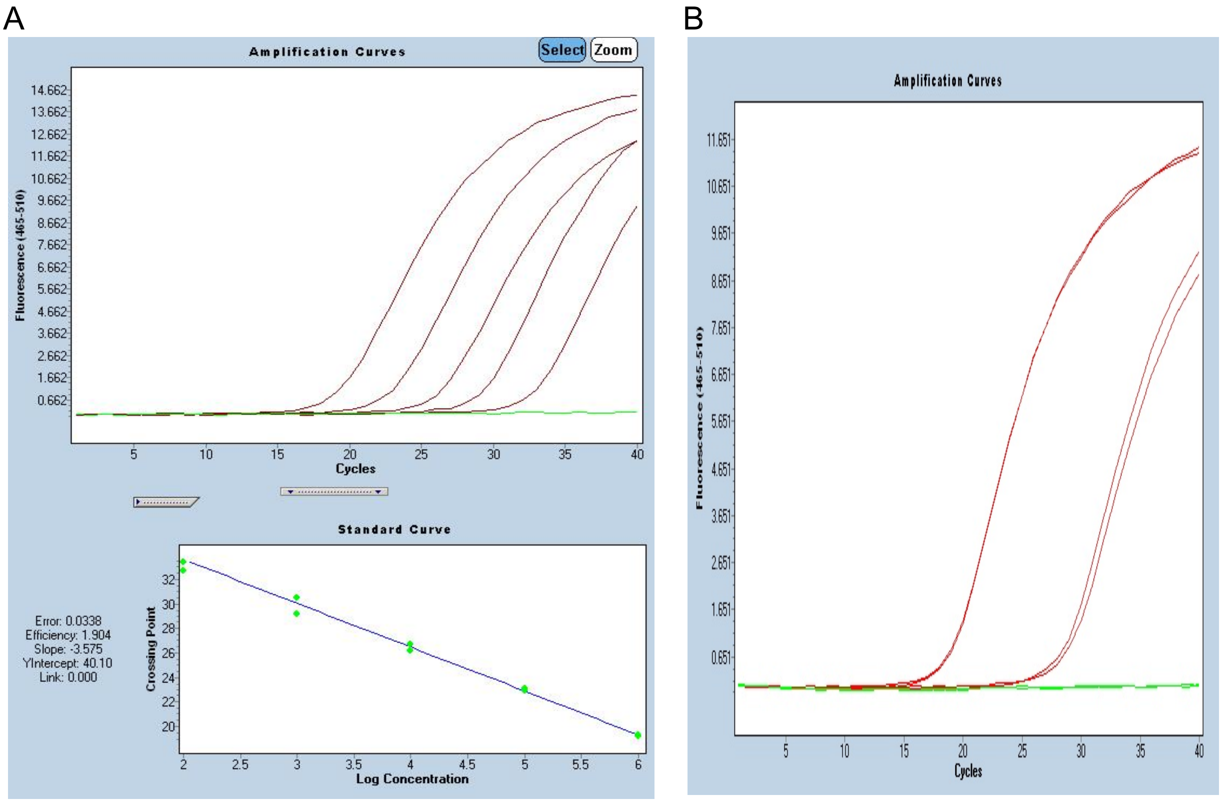

Figure 3S: ASB-PCR assay (Mut2.1) for detection of mutant KRAS G12D RNA. A) Representative qPCR amplification curves of serial dilution and linear regression analysis showing a compromised PCR efficiency of only 90%. B) qPCR amplification curves derived from the same copy number of either mutant transcript (left curves) or wildtype transcript (right curves).

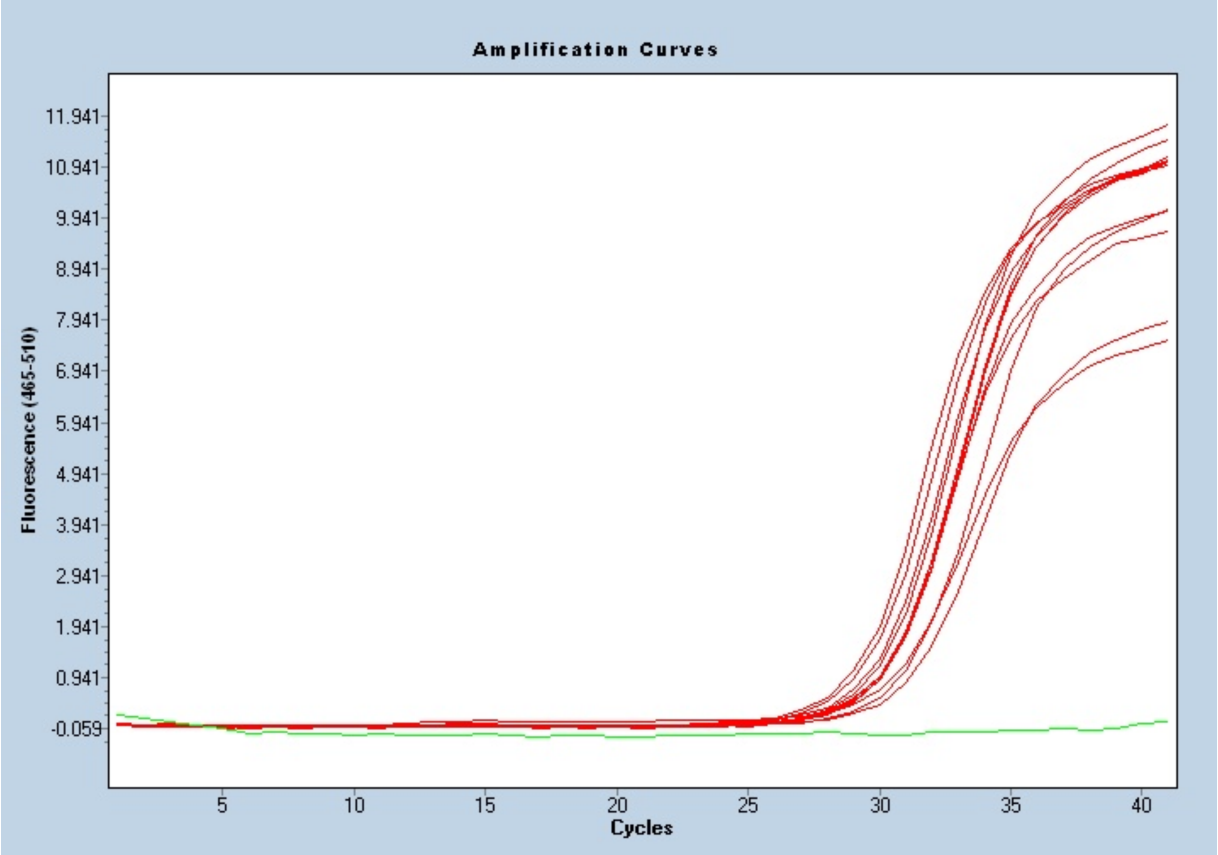

Figure 4S: Relative quantification of total amount of KRAS RNA from different clinical samples. Total amount of KRAS RNA from each clinical sample was quantified using a qPCR assay targeting a fragment upstream of the mutation site, which amplifies both the mutant and wildtype cDNA resulted from ExBP-RT assay. All tested samples showed comparable amount of total KRAS RNA with the Ct values ranging from 29.0 to 30.7 (0.6 – 1.8 x 10^5 copies/500ng of total RNA), in which the Ct value corresponding to the tested mutation-positive sample is 29.8. Primer and probe sequences and their concentration used in this qPCR assay are: KRAS-Up/Fw: 5' - AGGCCTGCTGAAAATGACTG -3'(0.6 μM); KRAS-Up/Rv: 5' - GCTCCAACTACCACAAG – 3'(0.6 μM); and KRAS-Up/P : FAM-AGAGGCTCAGCGGCTCCCAG – BHQ1 (0.4 μM)

1	2	3	4	5	6	7	8	9	10	11	12	13	14	15
50 bp Ladder	**blank**	**Pos ctr**	**H_2O RT**	#1	#2	#3	#4	#5	#6	#7	#8	#9	#10	#11

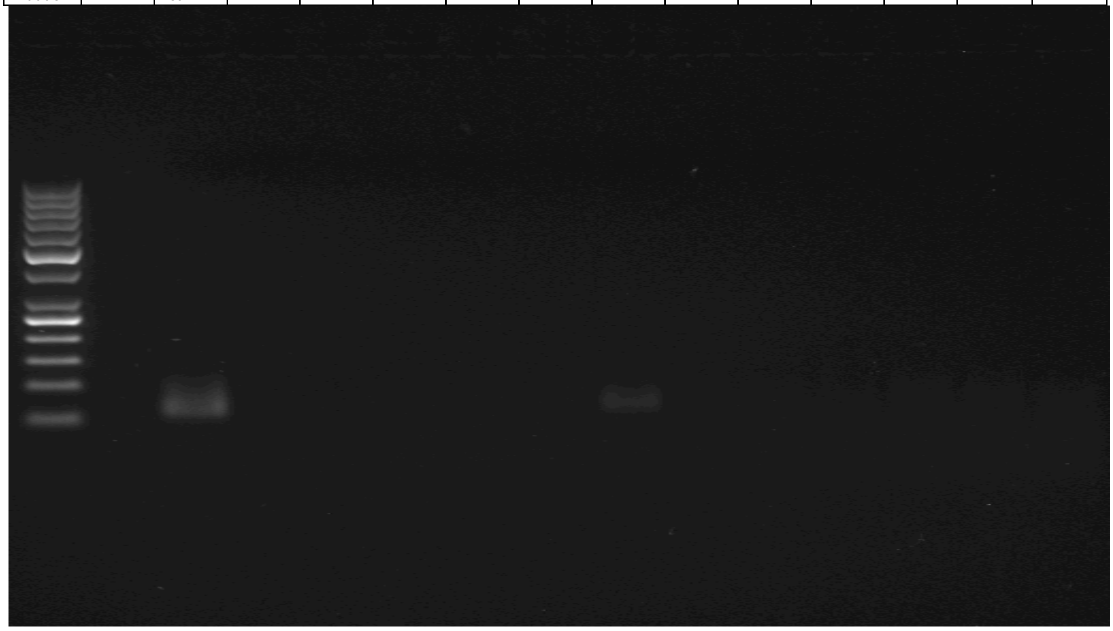

Figure 5S. Visualization of ExBP-RT amplification products derived from FFPE clinical samples on agarose gel 2%. The expected size (59 bp) of specific amplified sequences has been confirmed for both the positive control (lane 3) and the positive-tested sample (lane 9 - sample #5). No specific amplification product has been detected on gel for all negative-tested samples (sample #1-4 and #6-11) as well as the negative control without template. Lanes 1 is GeneRuler 50bp DNA ladder, ready-to-use (Thermo Scientific). Lanes 2 is blank well.

A.

B.

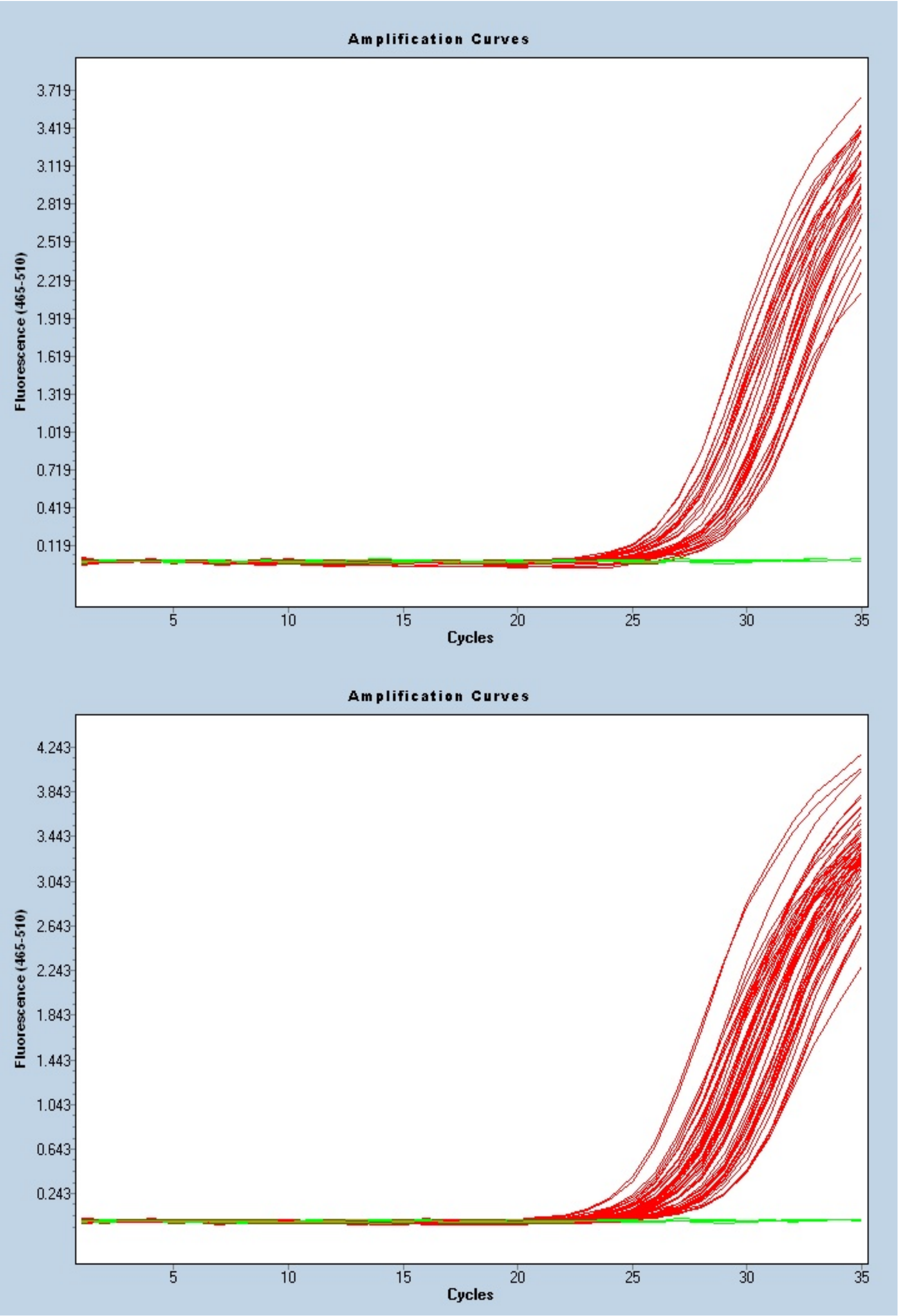

Figure 6S: Quantification of total KRAS RNA from different clinical samples in multiplex ExBP-RT assay. Total amount of KRAS RNA from each clinical sample was quantified using a qPCR assay targeting a fragment upstream of the mutation site, which amplifies both the mutant and wildtype cDNA resulted from ExBP-RT assay. All KRAS mutation positive-tested samples (A) and negative-tested samples (B) showed comparable amount of total KRAS RNA.

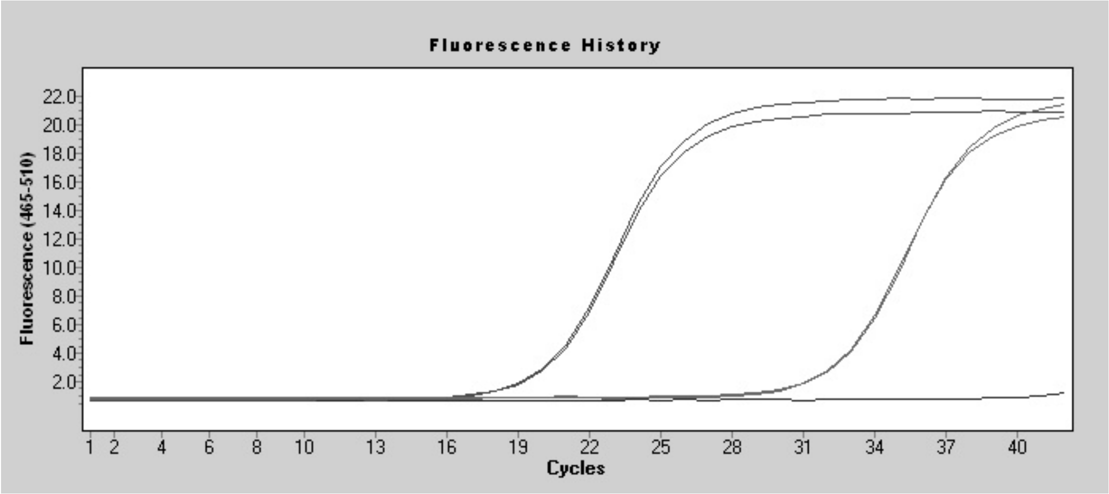

Figure 7S. qPCR amplification curves derived from the same copy number (10^8 copies) of either mutant KRAS G12D transcripts (left curves) or another mutant variant, KRAS G12A transcript as a wildtype surrogate (right curves). cDNA synthesis reactions were performed following the same protocol described in the Methods section. The sequence of the Extendable competitive Blocking Probe is 5'-GCCAGCAGC-3', which is fully complementary to the mutant KRAS G12A RNA. The resulting $\Delta Ct_{wt\text{-}mt}$ of this ExBP-RT assay is 11.01 cycles, corresponding to an excellent selectivity of 0.05%.
